# Effect of the Cationic Block Structure on the Characteristics of Sludge Flocs Formed by Charge Neutralization and Patching

**DOI:** 10.3390/ma10050487

**Published:** 2017-05-03

**Authors:** Huaili Zheng, Li Feng, Baoyu Gao, Yuhao Zhou, Shixin Zhang, Bingchen Xu

**Affiliations:** 1 Key Laboratory of the Three Gorges Reservoir Region’s Eco-Environment, Ministry of Education, Chongqing University, Chongqing 400045, China; zhl@cqu.edu.cn (H.Z.); zyh910531@126.com (Y.Z.); Zhangshixin2000@163.com (S.Z.); XuBC2008@163.com (B.X.); 2 Shandong Key Laboratory of Water Pollution Control and Resource Reuse, School of Environmental Science and Engineering, Shandong University, Jinan 250100, China; baoyugaosdu@yahoo.com.cn; 3 National Centre for International Research of Low-carbon and Green Buildings, Chongqing University, Chongqing 400045, China

**Keywords:** microblock structrue, cationic polyacrylamide, floc breakage and regrowth, fractal dimension, charge neutralization and patching

## Abstract

In this study, a template copolymer (TPAA) of (3-Acrylamidopropyl) trimethylammonium chloride (AATPAC) and acrylamide (AM) was successfully synthesized though ultrasonic-initiated template copolymerization (UTP), using sodium polyacrylate (PAAS) as a template. TPAA was characterized by an evident cationic microblock structure which was observed through the analyses of the reactivity ratio, Fourier transform infrared spectroscopy (FTIR), ^1^H (^13^C) nuclear magnetic resonance spectroscopy (^1^H (^13^C) NMR), and thermogravimetry/differential scanning calorimetry (TG/DSC). The introduction of the template could improve the monomer (AATPAC) reactivity ratio and increase the length and amount of AATPAC segments. This novel cationic microblock structure extremely enhanced the ability of charge neutralization, patching, and bridging, thus improving the activated sludge flocculation performance. The experiments of floc formation, breakage, and regrowth revealed that the cationic microblock structure in the copolymer resulted in large and compact flocs, and these flocs had a rapid regrowth when broken. Finally, the larger and more compact flocs contributed to the formation of more channels and voids, and therefore the specific resistance to filtration (SRF) reached a minimum.

## 1. Introduction

In sludge conditioning, flocculation is regarded as an important process in which the colloidal and suspended particles are separated and removed subsequently [1]. Three main flocculation mechanisms, namely, bridging, charge neutralization, and patching, play a critical role in the flocculation process [2]. For the bridging mechanism, the polymers adsorb on the particles surface and their loops and tails will attach themselves to bare patches on the approaching particles to form bridges, thus causing them to aggregate [3]. Generally, the molecular weight (MW) and chain length of the polymer are considered as the dominated factors for bridging. However, the situation of charge neutralization and patching are different from that of bridging. Previous studies have demonstrated that electrostatic interaction not only contributed to a strong adsorption, but also neutralized the negative charge completely [4]. Thus, flocculation occurred as a result of the reduced surface charge of the particles and the decreased electrical repulsion between them [5]. Therefore, the flocculant with high charge density (CD) has a strong charge neutralization ability and it is especially effective to form large flocs as the molecular weight (MW) of the flocculant is not too high [6]. When the average distance between particle surface sites is greater than that between charged segments in the polymer chain, electrostatic patch flocculation occurs because the adsorption of the polymer onto local sites of a particle causes a patch of local charge reversal and results in a positive-negative attraction between the particles [7]. Because the sludge particles are negatively charged, the charge neutralization mechanism becomes more and more important. It is generally accepted that the polymer with a strong charge neutralization ability will perform well in sludge flocculation [8]. Therefore, the research and development of a high-efficiency flocculant with a strong charge neutralization ability is very crucial in sludge flocculation.

**Figure 1:**
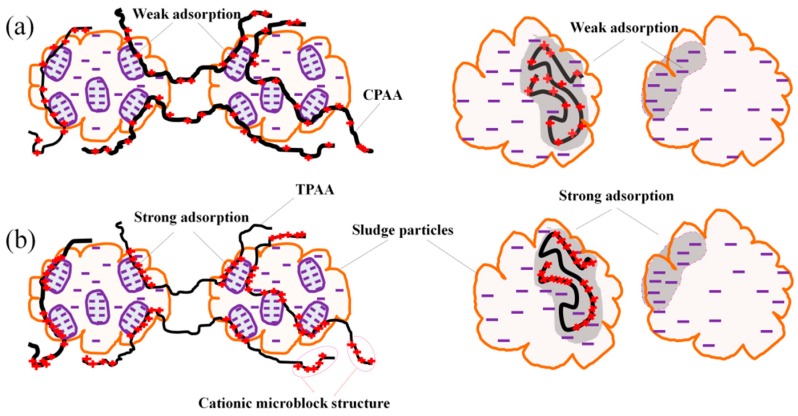
The charge neutralization and patching models for the polymer with (**a**) random distribution of cationic monomers and (**b**) cationic microblock structure. CPAA: non-template copolymer of (3-Acrylamidopropyl)). TPAA: template copolymer of (3-Acrylamidopropyl).

Recently, it has been found that the charge neutralization ability of the polymer was related to the sequence and distribution of cationic groups [9]. There is a drawback for the traditional polymers. The random distribution of cationic units in the polymer seriously hampers the further improvement of charge neutralization ability in the flocculation process. As shown in Figure 1a, the disordered cationic structure will waste a part of the positive charge, and hence the negative charged particles can-not be neutralized completely. Consequently, the flocculation efficiency is discounted greatly. On the contrary, if the polymer has a cationic microblock structure as shown in Figure 1b, the ability of charge neutralization and patching will be greatly enhanced and more negative charged particles will be strongly adsorbed to form large and compact flocs. However, it is not possible for the traditional methods to synthesize the flocculant with the cationic microblock structure. 

**Figure 2:**
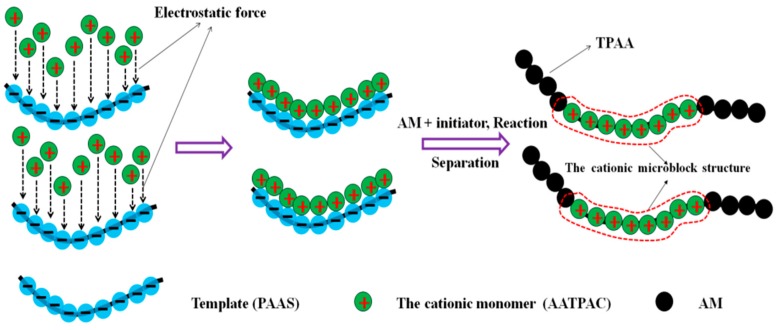
The proposed reaction route for the TPAA [template copolymer of (3-Acrylamidopropyl) trimethylammonium chloride (AATPAC) and acrylamide (AM)] with the cationic microblock structure.

Fortunately, the template polymerization technology can be used to synthesize the novel microblock structure in the polymer chain [10,11,12]. Its basic principle is shown in Figure 2. Prior to the copolymerization, an anionic template named sodium polyacrylate (PAAS) is added into the reaction system and the cationic monomer is adsorbed and arranged on the molecular chain of the template under the electrostatic force to form the as-prepared microblock structure. Once the copolymerization reaction is initiated by the initiator (VA-044), the pre-adsorbed cationic monomer will be homopolymerized to form the cationic microblock structure. After the separation of the template, a template copolymer with a novel cationic microblock structure is successfully synthesized. Although the improvement of this new method is not remarkable, the meaning for polymer science development and polymer applications is significant. The cationic microblock structure will enhance the positive charge density as well as the utilization efficiency, thus strengthening the ability of charge neutralization and patching. Meanwhile, the strong charge repulsion caused by the cationic microblock structure is more favorable for the extension of the polymer chain and thereby the bridging ability will be indirectly increased [13]. With the guidance of the template polymerization technology, (3-Acrylamidopropyl) trimethylammonium chloride (AATPAC) and acrylamide (AM) serve as monomers to prepare the novel template copolymer through the ultrasonic-initiated template copolymerization method (UTP). Undoubtedly, it will be more promising to use this new method to prepare polymers with high flocculation efficiency. 

In this study, the template copolymer (TPAA) of AATPAC and AM was prepared by UTP and its properties were analyzed by different advanced instruments such as FT-IR, ^1^H (^13^C) NMR and Thermogravimetric Analysis (TGA). Furthermore, to deeply understand the rules of template copolymerization, the reactivity ratios and sequence distribution of the monomers were statically analyzed. The effect of the cationic microblock structure on the floc size, morphology, breakage and subsequent regrowth, fractal dimension and specific resistance to filtration (SRF) was also investigated. Finally, the mechanism involved in the flocculation test was discussed and determined. 

## 2. Materials and Methods

### 2.1. Materials

The cationic monomer AATPAC (74–76 wt % in water) (Longsheng Chemical Co., Ltd., Shanghai, China). AM, Urea [CO(NH_2_)_2_], and template sodium polyacrylate (PAAS) with a molecular weight (MW) of 4200 (Lanjie Tap Water Company, Chongqing, China). Initiator 2,2′-azobis [2-(2-imidazolin-2-yl) propane] dihydrochloride (VA-044) (Apotheker Chemical Reagent Co., Ltd., Chengdu, China). AM is of technical grade, whereas the other reagents are of analytical grade. Commercial flocculants CPDA and CPMA were selected to make a comparison in flocculation tests. CPDA was the copolymer of AM and acryloyloxyethyl trimethyl ammonium chloride (DAC), and CPMA was prepared by AM and methacryloxyethylt rimethyl ammonium chloride (DMC).

### 2.2. Synthesis and Characteristics of Copolymers

**Scheme 1:**
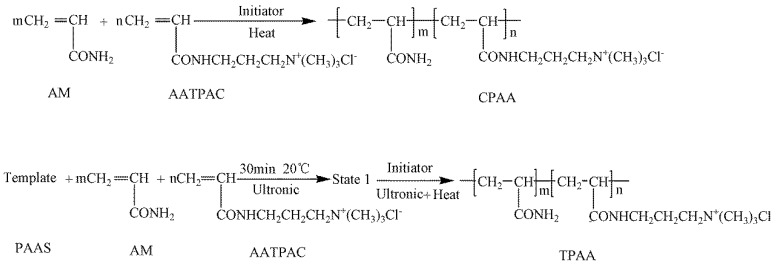
The proposed reaction routes for CPAA [non-template copolymer of (3-Acrylamidopropyl) trimethylammonium chloride (AATPAC) and acrylamide (AM)] and TPAA.

The ultrasonic-initiated template copolymerization method was employed to synthesize the template copolymer TPAA, whereas the preparation of non-template copolymer CPAA was similar to that of TPAA except that no template or ultrasonics were used. Both the template copolymer TPAA and no template CPAA displayed a hydrophilic property and a good solubility. The details of the preparation of TPAA and CPAA are illustrated in the Supporting Text S1, and the proposed reaction routes for CPAA and TPAA are shown in Scheme 1. After the products were purified and dried completely, their properties were characterized by FT-IR, ^1^H (^13^C) NMR and thermogravimetry/differential scanning calorimetry (TG/DSC), and the details about those analytical methods are described in the Supporting Text S2. The intrinsic viscosities of the copolymers were measured by an Ubbelohde viscosity meter (Shanghai Shenyi Glass Instrumental Co. Ltd., China) and the cationic degree (DC) of those were calculated by the colloid titration method [14,15].

### 2.3. Determination of theMonomer Reactivity Ratio and Composition

As an important index parameter, the monomer reactivity ratio was investigated to understand the composition and sequence distribution of the polymers. The monomer reactivity ratio was calculated by Formula (1):

(1) $$r_{1}=\frac{k_{11}}{k_{12}}$$

where *r*_1_ was the monomer reactivity ratio, and *k*_11_ and *k*_12_ were the homopolymerization and copolymerization rate constant, respectively. The method for determining *r*_1_ is described in the Supporting Text S3 and the statistical results are shown in the Supporting Tables S1 and S2. If the monomer reactivity ratio value became much larger, it meant that the monomer was more likely to be homopolymerized rather than copolymerized. Meanwhile, the corresponding composition was determined by Formulas (2) and (3):

(2) $$F_{1}=\frac{r_{1}f_{1}^{2}+f_{1}(1-f_{1})}{r_{1}f_{1}^{2}+2f_{1}(1-f_{1})+r_{2}{\left(1-f_{1}\right)}^{2}}$$

(3) $$F_{2}=1-F_{1}$$

where *F*_1_ was the molar fraction of monomer AM in the copolymer and *F*_2_ was that for AATPAC, *r*_1_ and *r*_2_ were the reactivity ratio of AM and AATPAC, respectively, and *f*_1_ was the molar fraction of AM to the total monomers (AM and AATPAC) before the copolymerization. In this experiment, the *f*_1_ was from 0.1 to 0.9.

### 2.4. Determination of the Sequence Distributions

Based on the monomer reactivity ratio, the average segment length and sequence distributions of the monomer segments were statistically analyzed. The microblock structure could be evaluated by the average segment length of the monomers, and the calculation equations were expressed as follows:

(4) $${\bar{N}}_{AM}=1+r_{AM}\frac{\left[M_{AM}\right]}{\left[M_{AATPAC}\right]}$$

(5) $${\bar{N}}_{AATPAC}=1+r_{AATPAC}\frac{\left[M_{AATPAC}\right]}{\left[M_{AM}\right]}$$

Here ${\bar{N}}_{AM}$ and ${\bar{N}}_{AATPAC}$ were the average segment lengths of the monomer AM and AATPAC, respectively. Meanwhile, *r_AM_* was the reactivity ratio of AM, and *r_AATPAC_* was that of AATPAC. [*M_AM_*] and [*M_AATPAC_*] were the dosages (unit: mol) of AM and AATPAC, respectively, before the reaction, and the mole ratio of [*M_AM_*] (0.060 mmol) to [*M_AATPAC_*] (0.040 mmol) was 1.500. The calculation results are shown in Supporting Table S3. Finally, the sequence distributions of the monomer segments were statistically analyzed by the following equations:

(6) $$\left(p_{1}{)}_{x}=p_{11}^{x-1}(1-p_{11}\right)$$

(7) $$p_{11}=\frac{r_{AM}\left[M_{AM}\right]}{r_{AM}\left[M_{AM}\right]+\left[M_{AATPAC}\right]}$$

(8) $$\left(p_{2}{)}_{x}=p_{22}^{x-1}(1-p_{22}\right)$$

(9) $$p_{22}=\frac{r_{AATPAC}\left[M_{AATPAC}\right]}{r_{AATPAC}\left[M_{AATPAC}\right]+\left[M_{AM}\right]}$$

where *x* was the number of the AM or AATPAC units in the corresponding segment, and (*p*_1_)*_x_* or (*p*_2_)*_x_* was the probability that *x* AM or *x* AATPAC units link together in turn, respectively. The *p*_11_ was the probability of only one AM unit in the polymer and *p*_12_ was that of two AM units linking together in the polymer. [*M_AM_*], [*M_AATPAC_*], *r_AM_* and *r_AATPAC_* were the same as those described in Equations (4) and (5). The results of the sequence distributions of the monomer segments are shown in Supporting Table S4.

### 2.5. Flocculation Tests

#### 2.5.1. Sludge and Flocculants

The details of the flocculants used for flocculation tests are listed in the Supporting Table S5. Municipal sludge was sampled from the Jiguanshi Wastewater Treatment Plant (Chongqing, China) with a cyclic-activated sludge system, and the characteristics of the sludge are listed in Table 1. 

#### 2.5.2. Jar Test

To determine the optical dosage for each flocculant, the specific resistance to filtration (SRF) was chosen as the evaluation index. The jar test was performed on a program-controlled Jar-test apparatus (ZR4-6, Zhongrun Water Industry Technology Development Co. Ltd., Shanghai, China) at room temperature. The jar test included an initial rapid stirring at 200 rpm for 1 min, a slow stirring at 40 rpm for 15 min, and a setting period of 20 min [16]. During the rapid stirring of 200 rpm, a given dosage of the flocculant was added to a 1.4 L beaker with a 1.0 L of sludge, and the dosage ranged from 10 to 90 mg∙L^−1^. Each jar test was repeated three times and the final results were averaged. The method for the SRF is illustrated in Supporting Text S4 and the results are shown in Supporting Figure S2. It was found that the lowest SRF for each flocculant was reached at the dosage of 40 mg∙L^−1^. The zeta potential of the supernatant before breakage was measured by a Zetasizer Nano ZS90 (Malvern Instruments Ltd., Malvern, UK).

#### 2.5.3. Floc Formation, Breakage, and Regrowth

Flocculation tests for floc formation, breakage, and regrowth were carried out by a jar test as stated above. After the slow stirring period of 15 min, the aggregated flocs were exposed under the high shear force at 200 rpm for 5 min and were then followed by another 15 min of slow stirring at 40 rpm for floc regrowth [17]. During the whole process, the dynamic floc image was investigated though the in-site system which consisted of a computer-controlled digital camera (DP10, Olympus, Hong Kong, China), and the floc morphological parameters such as projected area, perimeter, and diameter were analyzed by image processing software (Image-Pro Plus, version 6.0, Media Cybernetic, Rockville, MD, USA) [18]. The details of the in-site system and the method for determination of the fractal dimension were illustrated in the previous study [19], and the dynamic floc size, fractal dimension, and the floc size after breakage were all obtained. The zeta potential of the supernatant and the SRF of the flocs after the breakage were also investigated to make a comparison with those before breakage.

## 3. Results and Discussion

### 3.1. The Monomer Reactivity Ratio and Composition

The monomer reactivity ratio and the composition of the polymer were calculated through the Kelen-Tüdӧs method and the composition equation, respectively. In Table 2, the *r_AATPAC_* for TPAA was much higher than that for CPAA, whereas the situation of r_AM_ was just the opposite. The addition of the template had a significant effect on the monomer reactivity ratios, for example, the *r_AATPAC_* increased from 0.358 (CPAA) to 0.751 (TPAA) and the r_AM_ declined from 2.815 (CPAA) to 1.586 (TPAA). The monomer reactivity ratio difference between TPAA and CPAA was related to their structural properties. During the process of template copolymerization, the cationic monomers (AATPAC) adsorbed on the surface of template molecular chain had more chance to be homopolymerized with each other, thus forming the evident AATPAC microblock structure. In this case, a part of AM with a higher monomer reactivity ratio will lose the chance of copolymerization with AATPAC and therefore the reactivity ratio of AM decreased.

**Figure 3:**
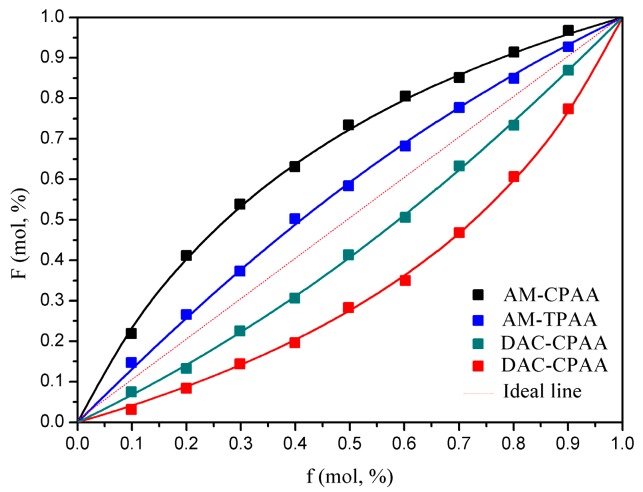
Composition curves of the polymers.

As shown in Figure 3, the composition curves of AM-TPAA and AATPAC-TPAA became much closer to the ideal line than those of AM-CPAA and AATPAC-CPAA, respectively. It indicated that the addition of template was more beneficial for improving the AATPAC conversion. The template was just like a protective shell in which the cationic monomer could be homopolymerized efficiently to form the microblock structure. The protective shell resulted in a big space steric hindrance, which would make the free collision reaction of AM become more and more inconvenient. As a result, the reactivity ratio of AM decreased.

### 3.2. The Sequence Distributions of the Polymers

**Figure 4:**
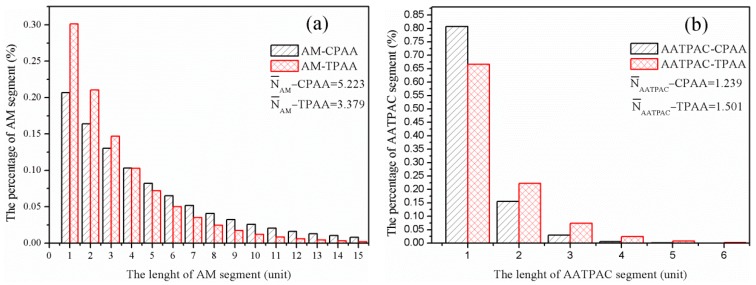
Sequence distributions of (**a**) acrylamide (AM) and (**b**) (3-Acrylamidopropyl) trimethylammonium chloride (AATPAC) in polymers.

The average length of the monomer segment was measured to confirm the formation of an evident cationic block structure (AATPAC microblock) in TPAA. According to the calculation results obtained from Equations (4) and (5), the ${\bar{N}}_{\mathrm{AATPAC}}$ in TPAA was much longer than that in CPAA, whereas the situation for ${\bar{N}}_{\mathrm{AM}}$ was the reverse. For instance, the value was 1.501 for ${\bar{N}}_{\mathrm{AATPAC}}$-TPAA, 1.239 for ${\bar{N}}_{\mathrm{AATPAC}}$-CPAA, 3.379 for ${\bar{N}}_{\mathrm{AM}}$-TPAA, and 5.223 for ${\bar{N}}_{\mathrm{AM}}$-CPAA. This indicated that more evident AATPAC microblocks were synthesized through template copolymerization, which was consistent with the analytical results of the monomer reactivity ratio and composition. Furthermore, the sequence distributions of the TPAA and CPAA were statistically calculated and the results are displayed in Figure 4. Obviously, the proportion of the long AATPAC segments (length > 1) in TPAA soared to 34.1%, compared with that in CPAA (18.2%). However, the ${\bar{N}}_{\mathrm{AM}}$-TPAA was shortened by the template addition. A similar phenomenon was found by previous research [21]. Because of the template skeleton effect and electrostatic force between the template and cationic monomers, AATPAC was tightly adsorbed and arranged on the template molecule chain to form the precursor of the microblock structure. During the process of reaction, the microblock structure would be integrally stable due to the template protection even under adverse chemical environments. When the free radical initiation reaction was finished, the AATPAC and AM microblock were regularly arranged on the TPAA molecular chain.

### 3.3. Characterization of the Flocculants 

#### 3.3.1. FT-IR Spectral Analysis

**Figure 5:**
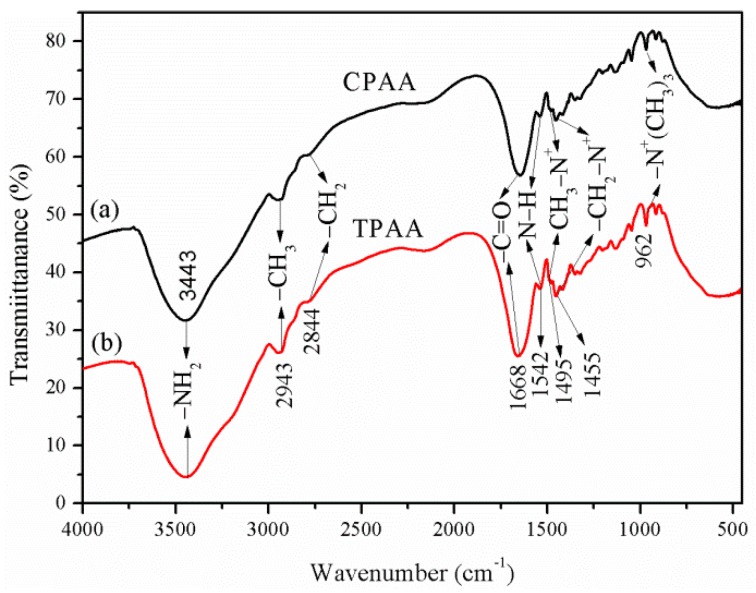
FT-IR spectra of (**a**) CPAA and (**b**) TPAA.

The FT-IR spectra of CPAA and TPAA are shown in Figure 5a,b, respectively. It was clear that the adsorption peaks of the two copolymers were almost the same, thus revealing that the template copolymer method had no impact on the chemical group structure of the copolymers. The adsorption bands at 3443 cm^−1^ and 1668 cm^−1^ were derived from −NH_2_ and C=O stretching vibration in AM, respectively. The asymmetric stretching vibrations of −CH_3_ (2943 cm^−1^) and −CH_2_− (2844 cm^−1^) were observed. Characteristic peaks of AATPAC at 1542, 1495, 1455, and 962 cm^−1^ correspond to −NH bending vibration of the mono-substituted amide group, −CH_3_ stretching vibrations in the −N^+^−(CH_3_)_3_ group, −CH_2_− bending vibrations in the −CH_2_−N^+^ group, and −N^+^−(CH_3_)_3_ bending vibrations, respectively [22,23]. The analytical results of the FT-IR spectra indicated that the TPAA and CPAA polymers were all successfully synthesized through copolymerization of AM and AATPAC.

#### 3.3.2. ^1^H NMR Spectral Analysis

**Figure 6:**
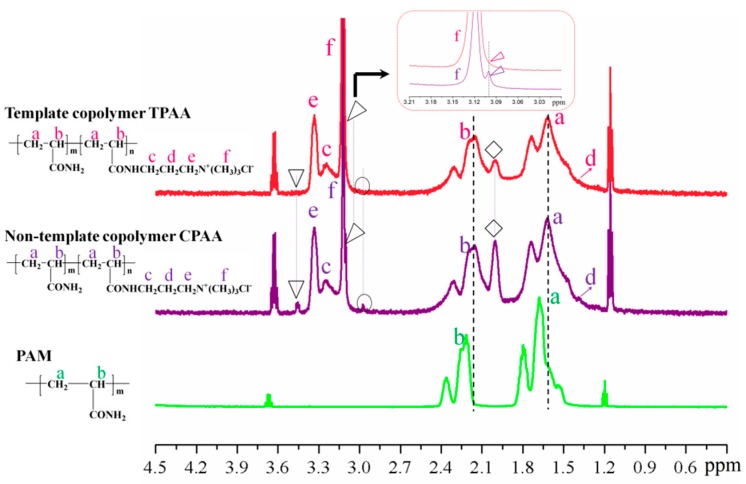
The ^1^H NMR spectra of the polymers.

The ^1^H NMR spectra of the copolymers were investigated and the results are given in Figure 6. The characteristic peaks of each polymer were all observed, which indicated that the polymers were all successfully synthesized. For example, the peaks at 1.624, 2.167, 3.246, 1.397, 3.328, and 3.120 ppm were assigned to the protons (H_a_-H_f_) in both TPAA and CPAA, respectively. [24] Compared with PAM, the chemical shifts of protons (H_a_ and H_b_) in TPAA and CPAA occurred. The chemical shifts of the protons were related with their chemical environment, and different chemical environments would result in different chemical shifts [24]. TPAA and CPAA were the copolymers of which the proton’s chemical environment was not the same with that of the homopolymer (PAM), and therefore their chemical shifts of the protons were not identical. Furthermore, the TPAA and CPAA were demonstrated to have many similarities, but several subtle differences were very significant to this study and should not be ignored. Three weak peaks in CPAA were observed at 3.447, 3.101, and 2.972 ppm, respectively, whereas those in TPAA disappeared. Meanwhile, the intensity of the peak at 2.004 ppm was strong in CPAA, but it became weak in TPAA. This interesting phenomenon could be explained as follows. The monomers (AM and AATPAC) were distributed randomly in the copolymer chain of CPAA, and the distribution of the monomers was disordered and uncontrollable. As a result, the proton’s chemical environment of the monomer AM or AATPAC became different and it was vulnerable to the adjacent protons. Thus, more split peaks of protons in CPAA were observed. However, the situation for TPAA was just the opposite. Because of the strong steric and electrostatic repulsion of the pendant groups that resulted from the evident microblock structure (AM and AATPAC segments) in TPAA, the proton’s chemical environment of the monomers became more identical rather than diverse [21,25]. Therefore, the protons in each chemical group would have only one peak in principle. For instance, the protons of H_e_ or H_f_ generated only one peak rather than two or three peaks. Although the peak at 2.004 ppm did not vanish in TPAA, the intensity of the peaks decreased sharply and the peak became much weaker, compared with that of CPAA. Based on the above analyses, it further indicated that the evident cationic microblock structure was formed in TPAA.

#### 3.3.3. ^13^C NMR Spectral Analysis

**Figure 7:**
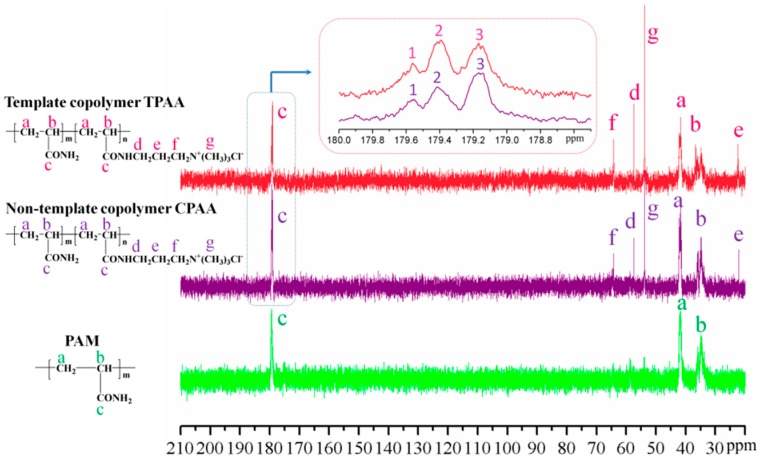
The ^13^C NMR spectra of the polymers.

Figure 7 displays the ^13^C NMR spectra of TPAA, CPAA, and PAM. It was clear that the ^13^C NMR spectra of TPAA and CPAA were also the same, but not identical. Compared with the ^13^C NMR spectra of PAM, the characteristic peaks (^13^C_d_-^13^C_g_) of TPAA and CPAA were all observed, thus indicating the successful formation of TPAA and CPAA [26]. Previous research has demonstrated that the proportion of the monomer segment could be obtained through the area calculation of the carbonyl carbon resonance peak [20]. If an AM unit was denoted as A and AATPAC unit was denoted as T, the triads were, in order from 1 to 3, AAA, AAT, and TAT [27]. The MestReNova software was employed to calculate the mole fraction of the triads’ sequence structure and the results are shown in Table 3. Obviously, the addition of the template resulted in a huge impact on the mole fraction of the triads. The mole fraction of the AAA decreased from 57.4% (CPAA) to 42.8% (TPAA), whereas those of AAT and TAT increased from 36.3% and 6.3% in CPAA to 48.1% and 9.1% in TPAA, respectively. Due to the formation of the evident cationic microblock in TPAA, the AATPAC segment would show an increase in the mole fraction as well as the average length. Meanwhile, the reactivity of AM homopolymerization would be limited by the template, and therefore the mole fraction of the AAA and the average length of the AM segment declined. The ^13^C NMR analytical results of the polymers indicated that the evident AATPAC segment was successfully synthesized in TPAA rather than CPAA, which corresponded with the results of the sequence distributions of polymers shown in Section 3.2. 

#### 3.3.4. Thermogravimetry/Differential Scanning Calorimetry Analysis

**Figure 8:**
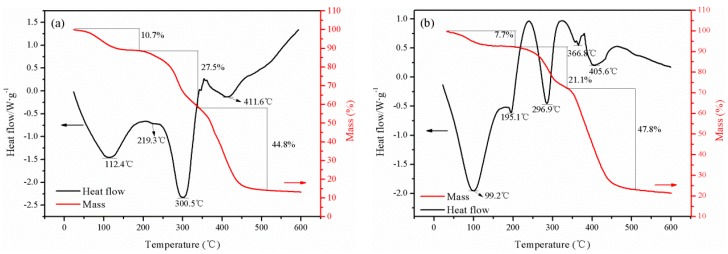
Thermogravimetry/differential scanning calorimetry (TG/DSC) analysis of (**a**) CPAA and (**b**) TPAA.

The microstructures of TPAA and CPAA were investigated by the Thermogravimetry/differential scanning calorimetry (TG/DSC) analysis and the thermal gravimetric curves are shown in Figure 8. The two copolymers displayed three main stages of thermal decomposition with their weight loss. In the first stage, the mass loss was about 10.7 wt % for CPAA in the range of 30–220 °C and 7.7 wt % for TPAA in the range of 30–195 °C, which was assigned to the evaporation of intramolecular and intermolecular moisture in the polymers [28]. In the second stage, mass loss was about 27.5 wt % for CPAA in the range of 220–350 °C and 21.1 wt % for TPAA in the range of 195–350 °C, which was due to the imine reaction of the amide group and the thermal decomposition of methyl in the quaternary ammonium groups [29]. The final stage occurred above 350 °C, and the mass loss was about 44.8 wt % for CPAA while it was about 47.8 wt % for TPAA, which was ascribed to the carbonization of the copolymer. In the final stage, two evident heat absorption peaks at 366.8 °C and 405.6 °C in the DSC curve of TPAA were observed, whereas only one peak at 411.6 °C appeared in the DSC curve of CPAA. It was supposed that this interesting phenomenon was related to the special microblock structure in the polymer chain. TPAA with the AM and AATPAC microblock structure would generate two evident heat absorption peaks. However, it was impossible for CPAA to form two microblock structures because of the random distribution of the monomers in its polymer chain. Therefore, only one heat absorption peak for TPAA could be observed. A similar finding was reported by previous studies [9,20]. Thus, the results of the TG/DSC analysis provided further support for the formation of the microblock structure in TPAA.

### 3.4. Flocculation Performance

#### 3.4.1. Zeta Potential 

**Figure 9:**
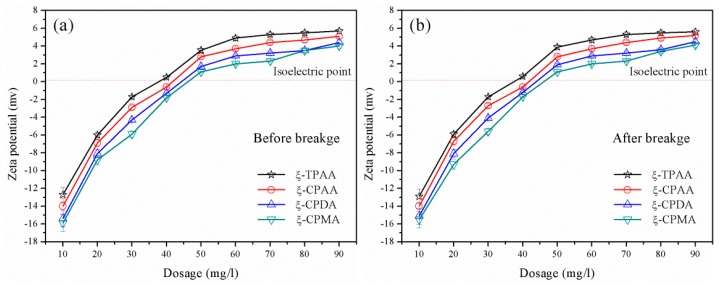
Zeta potentials of the flocs as a function of the coagulant dosage before (**a**) and after (**b**) breakage.

The ability of charge neutralization and patching was evaluated by the zeta potential [30]. When the flocculants with the same intrinsic viscosity and cationic degree were used in the sludge flocculation, the charge neutralization and patching became dominant and the negatively charged particles were neutralized completely, thus aggregating and forming large flocs. The flocculant with the strong charge neutralization and patching ability was more favorable for the excellent flocculation performance occurrence. Therefore, the determination of the zeta potential was more vital and meaningful for the flocculant. As shown in Figure 9, the zeta potentials of the supernatant conditioned with the flocculants all rapidly increased at a dosage ranging from 10 mg L^−1^ to 40 mg∙L^−1^ and then gradually climbed up a plateau at a dosage ranging from 40 mg L^−1^ to 90 mg∙L^−1^. However, the zeta potential for TPAA was the highest for all the flocculants, which indicated that the charge neutralization and patching ability of the TPAA was the strongest. Due to the cationic microblock structure, namely, the AATPAC microblock structure in the TPAA, the distribution of the positive charge became more intensive rather than random for the other three (CPAA, CPDA and CPMA), and therefore TPAA showed the strongest charge neutralization and patching ability. Meanwhile, the dosage of the TPAA at the isoelectric point was the lowest among them, which also revealed that TPAA had an obvious advantage in charge neutralization and patching. The zeta potentials were also the same before and after the breakage, which indicated that the vigorous stirring had no impact on the zeta potential. This similar phenomenon has been found in previous research [31].

#### 3.4.2. Floc Formation, Breakage, and Regrowth

**Figure 10:**
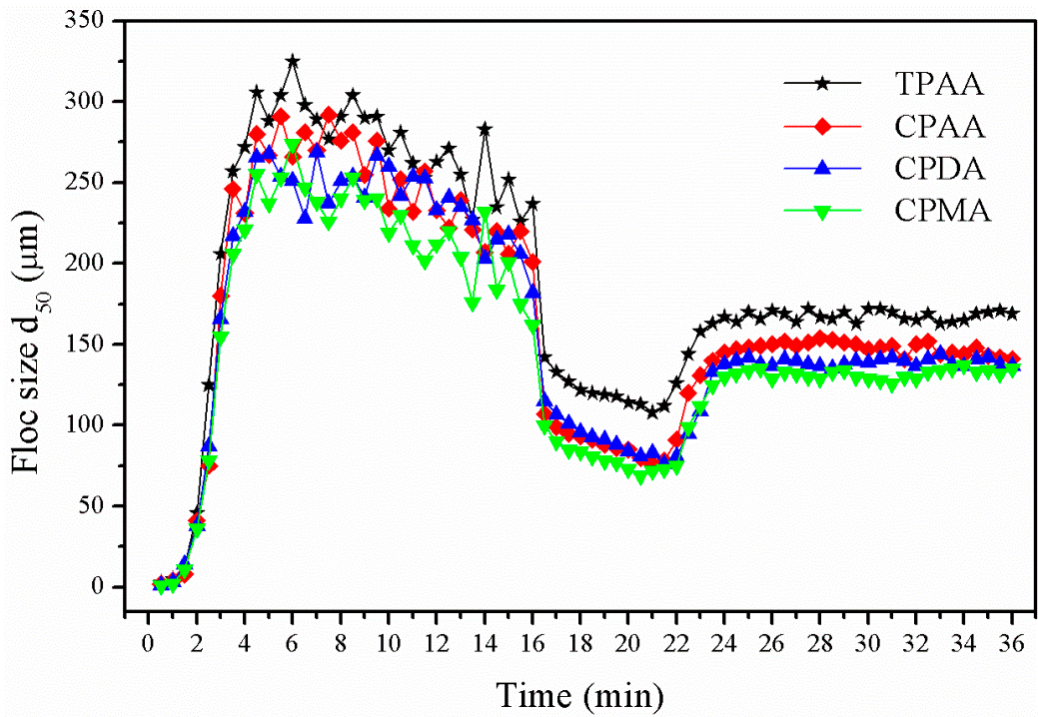
Floc formation, breakage (5 min of 200 rpm) and regrowth process with different flocculants.

Floc formation, breakage, and regrowth with different flocculants were monitored as flocculation proceeded. The variation of the median equivalent diameter (d_50_) of the floc size versus flocculation time was displayed in Figure 10. It was found that the variation trend was similar for the flocculants, while the extent of the breakage and regrowth of flocs were different under the same dose and intrinsic viscosity. The floc size increased rapidly for all four flocculants and reached a plateau during the slow mixing state. When the floc was exposed to a vigorous stirring of 200 rpm for 5 min, its size showed an immediate and sharp decrease, which indicated that the increased shear force led to a severe floc breakage. When the shear was removed, the flocs began to regrow to a steady-state size, implying a balance between the floc breakage and growth reached [32]. It was evident that it was hard for the floc to fully regrow to their original steady-state size after breakage regardless of any flocculants, thus indicating that some special combined bonds generated by charge neutralization and bridging rather than physical bonds between the particles and the flocculant were destroyed under the high shear force [33]. However, compared with the other three flocculants, TPAA showed a significant improvement in floc reformation and regrowth and the flocs conditioned by TPAA had a strong recoverability. It was demonstrated that the floc size with TPAA in all cases was the largest among the four flocculants. It was suggested that the mechanisms involved in the flocculation were bridging, charge neutralization, and patching, and the charge neutralization and patching would be dominant as the bridging was limited or broken [34]. Because of the cationic microblock structure in TPAA, the charge neutralization ability and the electrostatic patching would be extremely enhanced. Once the floc was broken by the high shear force, the small fragments would be absorbed together to regrow a large floc. As a result, the recoverability of the floc was evidently improved by the cationic microblock structure in the flocculant.

#### 3.4.3. Fractal Dimension

**Figure 11:**
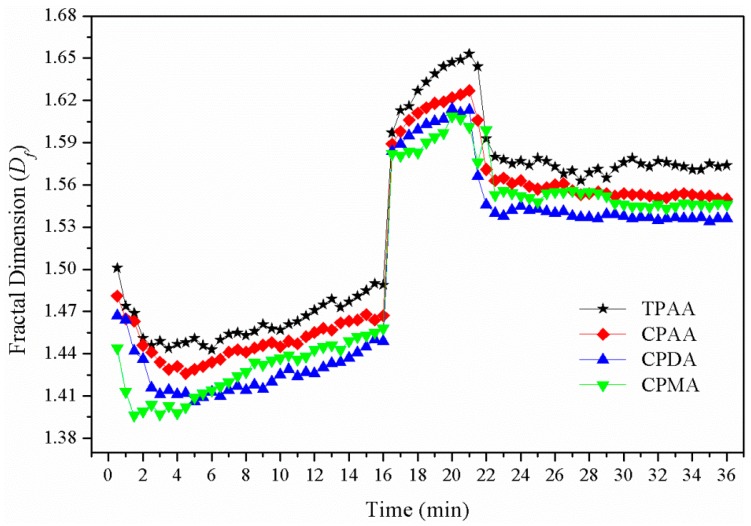
Fractal Dimension (D_f_) of the flocs with different flocculants as a function of time.

The fractal dimension (D_f_) was regarded as a vital parameter to evaluate the degree of compactness of the flocs, and it also had a strong influence on the solid/liquid separation efficiency [16]. A higher value of D_f_ represented a more compact floc structure and a higher effective density, and which was easier for solid setting and solid/liquid separation. As shown in Figure 11, the variation of the D_f_ versus flocculation time for different flocculants was investigated. The variation trend of D_f_ for the flocculants was similar, and it increased rapidly after breakage and then decreased to a steady-state during the regrowth state. It was obvious that TPAA had the highest D_f_ value among them in all cases, thus indicating that the flocs conditioned by TPAA were the most compact. It is worth noting that the D_f_ of TPAA showed a sharp increase during the breakage state. Before breakage, the cationic polyacrylamide with long and spiral chains was hydrolyzed in water and then the molecular chain would be unfolded and more functional groups were exposed, thus providing more chances to interact with particles to form large flocs. Although the bridging effect has contributed much to the large floc size, the floc was still not compact enough and sometimes presented a loose and porous floc structure [20]. When the higher shear force was introduced into this flocculation system, the flocs were broken at their weak points and re-arranged into more stable structures. Meanwhile, during the breakage process, some compact flocs located on the inner side of the porous flocs would be exposed and new chemical bonds would be rebuilt, and thereby their D_f_ value increased. However, the effects of charge neutralization and patching could not be ignored. The cationic microblock structure resulted in a strong interaction between the cationic group and the negative particles. This strong interaction was always due to some chemical bonds rather than physical bonds, which had a strong ability to resist the outside destruction, even if the chemical environment was severe. Once the steady-state flocs were broken, the destroyed fragments would be more likely to aggregate together to form a large and compact floc structure under the effects of strong charge neutralization and patching. The cationic microblock structure improved the floc structure, and a high value of D_f_ could be obtained.

#### 3.4.4. Floc Size Distribution

**Figure 12:**
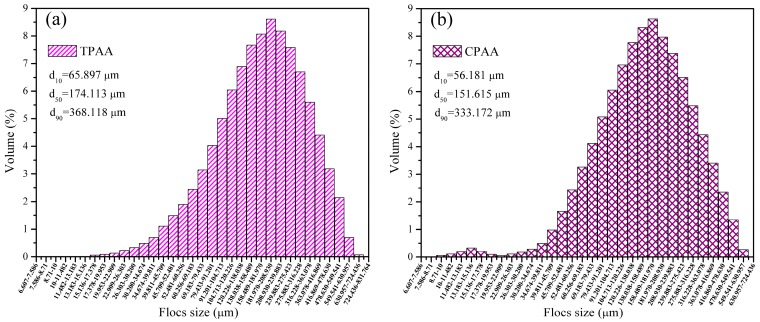

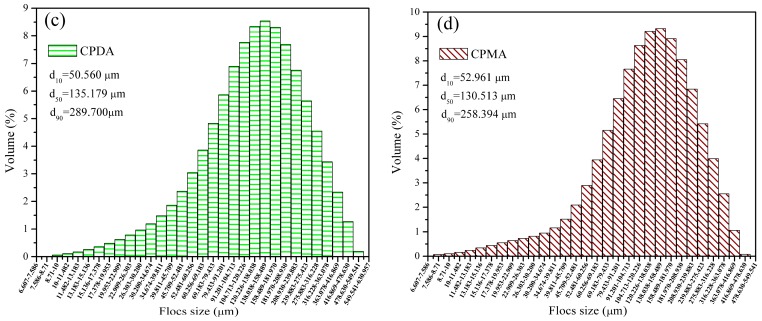
Floc size distribution for (**a**) TPAA; (**b**) CPAA; (**c**) CPDA and (**d**) CPMA.

Figure 12 displays the floc size distribution after the breakage stage for TPAA, CPAA, CPDA, and CPMA, respectively. Although the flocculants had the same intrinsic viscosity and cationic degree, the difference of their floc size distribution was evident. Compared with CPAA, CPDA, and CPMA, the d_10_ (10% in the range [0 μm-d_10_]), d_50_, and d_90_ (90% in the range [0 μm-d_90_]) of TPAA were the largest, respectively. Due to the cationic microblock structure in TPAA, the enlargement of the floc size was obtained, thus producing a positive effect on the sludge flocculation [35]. On the one hand, the strong electrostatic repulsion generated by the cationic AATPAC segment of TPAA would lead to a better expansion of its molecular chain, and the bridging ability would be improved. On the other hand, the cationic microblock structure had a strong charge density, demonstrated by Figure 9, and more negatively charged particles would be tightly absorbed on the TPAA polymer chain to form large and compact flocs [36]. When the flocs were exposed under the high shear force, the large and compact flocs still maintained a stable structure and the floc size of TPAA was still the largest among them. 

#### 3.4.5. Sludge Dewatering with the Floc Fractal Dimension

**Figure 13:**
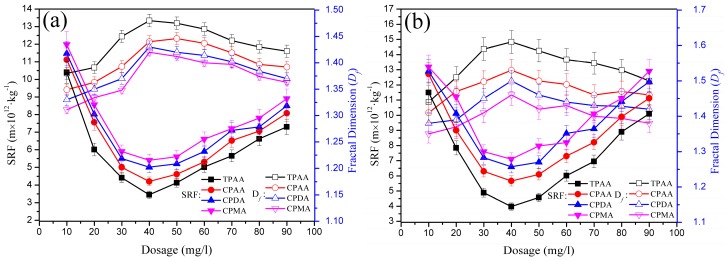
Sludge specific resistance to filtration (SRF) and floc fractal dimension (**a**) before and (**b**) after breakage.

Specific resistance to filtration (SRF) was an important index to assess the sludge filtration performance, and a lower value of SRF always indicated a better performance of sludge flocculation [37]. In Figure 13, the sludge SRF and fractal dimension (D_f_) before and after breakage were investigated. Clearly, the TPAA displayed the lowest SRF with the highest value of D_f_ among the flocculants in the full dosage range (10 to 90 mg∙L^−1^). For example, the minimum SRF (before breakage: 3.46 m × 10^12^∙kg^−1^; after breakage: 3.98 m × 10^12^∙kg^−1^) and the maximum D_f_ (before breakage: 1.48; after breakage: 1.61) for TPAA were acquired at the optimal condition (dosage: of 40 mg∙L^−1^). The TPAA with the cationic microblock structure had a strong charge attraction and neutralization ability, and thus the negatively charged particles would be attracted and neutralized completely, and then combined with the polymer chain tightly to form compact and large flocs. However, due to the random distribution of the cationic monomers in the polymer chain of the other three flocculants (CPAA, CPDA, and CPMA), the cationic microblock structure failed to form and the charge attraction and neutralization ability was discounted greatly, hence a loose and small floc structure was obtained. Previous studies have found that the SRF was related to sludge structure characteristics including fractal dimension (D_f_), floc size, and so on [19]. It was obvious that the D_f_ was linked with SRF, and the floc with a higher D_f_ reflected a lower SRF. The compact and large flocs accompanied with a relative high D_f_ were favorable for the sludge filtration. These large and compact flocs acting as a stable skeleton construction could withstand the high pressure test, and hence more and more stable channels and voids formed [38]. Therefore, it was easy for the water moisture to pass though the stable channels and voids, and the filtration resistance reached the minimum value. By contrast, the situation for the CPAA, CPDA, and CPMA was just the opposite. Because of the relatively loose and porous floc structure yielded by CPAA, CPDA, and CPMA, the pore canal for water discharge would shrink and even be closed under the high pressure, thus resulting in a large filtration resistance. The small flocs generated by CPAA, CPDA, and CPMA would plug the pore canal and deteriorate the filtration performance, and thereby a high SRF was observed. Based on the above analytical results, the cationic microblock structure contributed much to the low SRF and high D_f_, and therefore the sludge flocculation performance was greatly enhanced. The meaning of this novel flocculant with the microblock structure is significant and it will receive more and more attention in the application of sludge condition and dewatering.

### 3.5. Flocculation Mechanism

**Figure 14:**
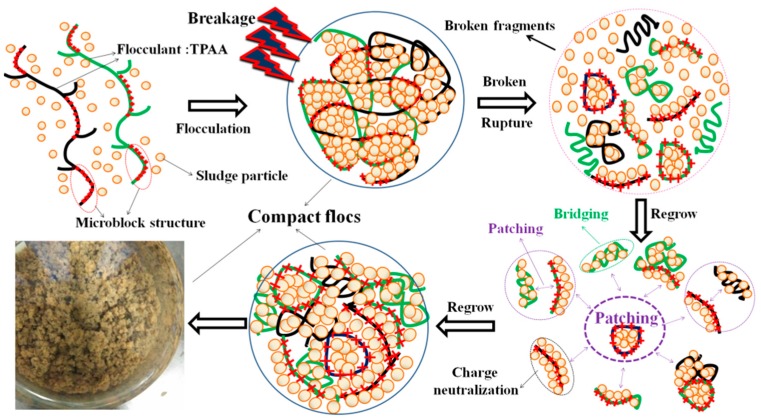
Possible flocculation mechanism of the TPAA.

Based on the analytical results of the zeta potential, floc formation, breakage and regrowth, D_f_ and SRF, the possible flocculation mechanism is summarized and displayed in Figure 14. In the entire flocculation process, the cationic microblock structure played an important role in strengthening the effect of bridging, charge neutralization, and patching. The intermolecular and intramolecular charge repulsion would be enhanced by the cationic microblock structure in TPAA, and therefore the molecular chain had a smooth extension and flexible configuration to reach a better bridging. The extended molecular chain would expose more active sites to capture more negatively charged particles to form large and compact flocs. Meanwhile, the charge neutralization and electrostatic patching mechanism involved in this flocculation contributed much to the rapid reformation of the broken floc fragments. When the flocs were destroyed by the high shear force, the broken fragments would rapidly aggregate to regrow the large and compact flocs under the driving force by electrostatic attraction. Therefore, the conclusion may be drawn that the chemical bonds generated by charge neutralization and patching were very stable and strong, and thereby had the advantages of being more insensitive to the high shear force. Even if the flocs were broken, the regenerative flocs still had a large size and compact structure.

## 4. Conclusions

In this study, a template copolymer TPAA with a cationic microblock structure was successfully synthesized though ultrasonic-initiated template copolymerization (UTP), and this novel microblock structure was confirmed by the analytical results of ^1^H (^13^C) NMR, TG/DSC, and the monomer reactivity ratio. Furthermore, the results of the flocculant tests proved that this novel microblock structure could lead to a significant improvement in sludge flocculation. The main conclusions were as follows: (1) The introduction of the template PAAS not only improved the monomer reactivity ratio of AATPAC, but also increased the number and the average length of the AATPAC segments, which was more favorable for the formation of an evident cationic microblock structure. Moreover, the addition of the template also increased the AATPAC conversion; (2) The cationic microblock structure of the polymer significantly improved the flocculation performance, and the flocculation test indicated that the cationic microblock structure in TPAA could help to generate a large and compact floc structure even under high shear force. Meanwhile, it also helped the broken floc segments to regrow rapidly when the floc was destroyed. Finally, this compact floc structure acted as a stable skeleton which was available for the formation of more stable channels and voids, and thereby the value of SRF declined; and (3) The mechanism involved in the flocculation tests was investigated, and the charge neutralization and patching were demonstrated to be the dominant mechanisms. The strong charge repulsion generated by the cationic microblock structure benefited the extension of the polymer chain, thus improving bridging. The chemical bonds coming from the charge neutralization and electrostatic patching were very stable and strong, and had a strong ability to resist the aggression of outside forces. Thus, a large and compact floc structure was acquired.

## Figures and Tables

**Table 1:** The characteristics of the sludge from the Jiguanshi Wastewater Treatment Plant.

pH	Zeta Potential (mV)	Mass Density (g·mL^−1^)	Moisture Content (%)	VSS/TSS
7.10	−22.30	1.038	0.996	0.771

^a^ VSS/TSS: Volatile suspended solids/Total suspended solid.

**Table 2:** The monomer reactivity ratios under n_PAAS_:n_AATPAC_ = 1:1 for TPAA and n_PAAS_:n_AATPAC_ = 0:1 for CPAA.

Kelen-Tüdӧs Method [20]		
Copolymer	*r*_AM_	*r*_AATPAC_
CPAA	2.815	0.358
TPAA	1.586	0.751

**Table 3:** The mole fraction of the triads’sequence structure.

MestReNova Software Method		
Copolymer	TAT (%)	AAT (%)
TPAA	9.1	48.1
CPAA	6.3	36.3

## Supplementary Materials

The following are available online at www.mdpi.com/1996-1944/10/5/487/s1.
