# Supplementary material for: Effect of the Cationic Block Structure on the Characteristics of Sludge Flocs Formed by Charge Neutralization and Patching
Source: Materials (Basel). 2017 May 3;10(5):487. doi: 10.3390/ma10050487 (PMC5459051; doi:10.3390/ma10050487)
Supplement: Supplementary file 1 [file materials-10-00487-s001.pdf]

Cover Page for Supporting Information:

Effect of the Cationic Block Structure on the Characteristics of Sludge Flocs Formed by Charge Neutralization and Patching

Huaili Zheng [1,3], Li Feng [1,3*], Baoyu Gao [2], Yuhao Zhou [1,3], Shixin Zhang [1,3], Bingchen Xu [1,3]

[1] Key Laboratory of the Three Gorges Reservoir Region's Eco-Environment, Ministry of Education, Chongqing University, Chongqing 400045,China; zhl@cqu.edu.cn (H.Z.); zyh910531@126.com (Y.Z.); Zhangshixin2000@163.com (S.Z.); XuBC2008@163.com (B.X.)

[2] Shandong Key Laboratory of Water Pollution Control and Resource Reuse, School of Environmental Science and Engineering, Shandong University, Jinan 250100, China; baoyugaosdu@yahoo.com.cn (B.G.) National Centre for International Research of Low-carbon and Green Buildings, Chongqing University, Chongqing 400045, China

* Correspondence: fl19860314@126.com; Tel./Fax: +86-23-6512-0827

Academic Editor: Christof Schneider

Received: 26 January 2017; Accepted:27 April 2017; Published: date

Total number of pages (including the cover page): 4 pages

This file contains 5 Tables, 4 Texts and 3 Figures.

Contents:

Text S1. The details of the preparation of TPAA and CPAA.

Text S2. The details of the FTIR, ^{1}H (^{13}C) NMR and TG/DSC.

Text S3. Kelen-Tüdös method.

Text S4. Analytical method for SRF.

Table S1. The calculating data relating to the monomer reactivity ratios in CPAA with the Kelen-Tüdös method under n_{PAAS}:n_{AATPAC}=0:1.

Table S2. The calculating data relating to the monomer reactivity ratios in TPAA with the Kelen-Tüdös method under n_{PAAS}:n_{AATPAC}=1:1.

Table S3. The average segment length of AM and AATPAC in CPAA and TPAA, respectively.

Table S4. The sequence distributions of the monomer segments.

Table S5. The details of flocculants used in the dewatering tests.

Fig. S1. Kelen-Tüdös Figure of AM and AATPAC reactivity ratio under (a) n_{PAAS}:n_{AATPAC}=0:1, (b) n_{PAAS}:n_{AATPAC}=1:1.

Fig. S2. Effect of the dosage on SRF.

Fig. S3. The ^{1}H NMR spectra of the CPMA and CPDA.

Text S1. The details of the preparation of TPAA and CPAA.

The preparation for TPTA and CPAA were as follows. First, a given amount of AM (60.00 mmol), AATPAC (40.00 mmol), urea (0.23 mmol), NaPAA (40.00 mmol; n_{NaPAA} : n_{TM} = 1:1), and deionized water (1.15 mol) was added into a 100 mL quartz jar. Second, the pH of the reaction solution was adjusted to about 4.5 by 0.5 mol L^{-1} HCl and NaOH, and then exposed under the ultrasonic wave radiation for 30 min to form a homogeneous solution at 20 °C. The ultrasonic wave was generated by an ultrasonicator (KQ 2200E, Kunshan ultrasonic instrument Co., LTD, China) and the frequency of that was 45 KHz. Third, the solution was deoxygenated through nitrogen bubbling for 30 min at ambient temperature. Finally, the quartz jar was sealed immediately after the addition of a given dose of initiator VA-044 to the mixture, and then continuously sonicated at 35-45 °C for 60-90 min. When the reaction was over, the copolymer was aged for 4 h at room temperature to increase the polymerization degree. Afterward, the copolymer was purified by acetone and dried until a constant weight was obtained. The preparation of CPAA was similar to that of TPAA except that no template and ultrasonication were used. The dried products were made into powder for the analyses of FTIR, ^{1}H (^{13}C) NMR, and TG/DSC.

Text S2. The details of the FTIR, ^{1}H (^{13}C) NMR and TG/DSC.

The FT-IR of the products were recorded on a 550 Series Ⅱ infrared spectrometer (Mettler Toledo Instruments Co., Ltd., Switzerland) using KBr pellets. The ^{1}H (^{13}C) NMR of the products were conducted on an Avance 500 nuclear magnetic resonance spectrometer (Bruker Company, Ettlingen, Germany) with deuterium oxide (D_2O) as the solvent. The thermal stabilities of the copolymers were investigated by TGA/DSC analysis which were carried out at a heating rate of 10 °C·min^{-1} under a nitrogen flow of 20 mL·min^{-1} from 20 to 600 °C on a DTG-60H synchronal thermal analyzer (Shimadzu, Kyoto, Japan).

Text S3. Kelen-Tüdös method

$$\eta = \left(r_{AM} + \frac{r_{AATPAC}}{\delta}\right)\xi - \frac{r_{AATPAC}}{\delta} \qquad (S3)$$

$$\eta = \frac{G}{H+\delta}\ ,\ \xi = \frac{H}{H+\delta}\ , \text{and}\ \delta = \sqrt{H_{max} \times H_{min}}\ ;\ H = \frac{R^2}{f}\ \text{and}\ G = \frac{R(f-1)}{f}$$

In the above formula, R is the molar ratio of the AM and AATPAC monomers in the

raw material before the copolymerization reaction, and f is the molar ratio of monomers in the polymers at a low conversion less than 15%. When the η and ξ of each point were obtained, the linear fitting curve relating to η and ξ could be plotted, and the r_{AM} and r_{AATPAC} could be obtained through the slope and intercept of the straight line. The calculating data relating to the monomer reactivity ratios with the Kelen-Tüdös method are displayed in Table S1 and Table S2. Finally, the monomer reactivity ratios were obtained, and r_{AM} (CPAA), r_{AATPAC} (CPAA), r_{AM} (TPAA), and r_{AATPAC} (TPAA) were 2.815, 0.358, 1.586 and 0.751, respectively.

Text S4. Analytical method for SRF.

SRF was calculated from Equation (1) [1]:

$$\mathrm{SRF} = \frac{2\mathrm{bpA}^2}{\mu \mathrm{c}} \qquad \text{Equation (1)}$$

where *SRF* is the specific resistance to filtration, *P* is the filtration pressure (N/m^2), *A* is the filtration area (m^2), μ is the viscosity of the filtrate (N·s/ m^2), *b* is the slope obtained from the plot of $t/V_f(y) - V_f(x)$, where V_f is the volume of filtrate (m^3) and *t* was the filtration time (s), and the filtrate volume was recorded at 5 s, 10 s, 20 s, 30 s, 40 s, 50 s, 60 s, 70 s, and 80 s during the filtration, and c was the weight of the solids per unit filtrate volume (kg/m^3), c= $[(1/C_i)/[(100C_i\text{-}C_f)/100C_f]$, where C_i was the initial moisture content (%) and C_f was the final moisture content (%).

Table S1

Table S1. The calculating data relating to the monomer reactivity ratios in CPAA with the Kelen-Tüdös method under n_{PAAS}:n_{AATPAC}=0:1

Number	R	*f*	G	H	ξ	η	δ
1	9.000 (9:1)	24.393	8.631	3.321	0.851	2.211	0.583
2	4.000 (8:2)	9.961	3.598	1.606	0.734	1.643	
3	2.331 (7:3)	5.522	1.908	0.983	0.628	1.218	
4	1.500 (6:4)	3.885	1.114	0.579	0.498	0.958	
5	1.000 (5:5)	2.831	0.647	0.353	0.377	0.690	
6	0.671 (4:6)	1.782	0.294	0.252	0.302	0.352	
7	0.432 (3:7)	1.243	0.084	0.149	0.203	0.115	

8	0.250 (2:8)	0.611	-0.160	0.102	0.149	-0.233	

Table S2

Table S2. The calculating data relating to the monomer reactivity ratios in TPAA with the Kelen-Tüdös method under n_{PAAS}:n_{AATPAC}=1:1

Number	R	*f*	G	H	ξ	η	δ
1	9.000 (9:1)	14.491	8.379	5.590	0.865	1.297	0.953
2	4.000 (8:2)	5.762	3.305	2.778	0.761	0.906	
3	2.331 (7:3)	3.938	1.738	1.379	0.613	0.773	
4	1.500 (6:4)	2.235	0.829	1.007	0.536	0.441	
5	1.000 (5:5)	1.331	0.248	0.752	0.463	0.153	
6	0.671 (4:6)	0.978	-0.015	0.459	0.345	-0.011	
7	0.432 (3:7)	0.675	-0.207	0.274	0.239	-0.180	
8	0.250 (2:8)	0.385	-0.399	0.162	0.157	-0.387	

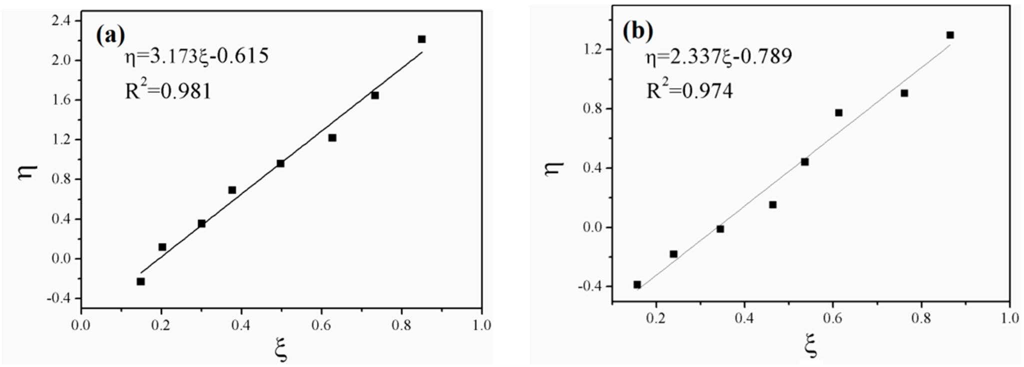

Figure. S1. Kelen-Tüdös Figure of AM and AATPAC reactivity ratio under (a) n_{PAAS}:n_{AATPAC}=0:1, (b) n_{PAAS}:n_{AATPAC}=1:1

Table S3.

The microblock structure could be evaluated by the average segment length of the monomers, and the calculation equations were expressed as follows:

$$\bar{N}_{AM} = 1 + r_{AM}\frac{[M_{AM}]}{[M_{AATPAC}]} \quad (4)$$

$$\bar{N}_{AATPAC} = 1 + r_{AATPAC}\frac{[M_{AATPAC}]}{[M_{AM}]} \quad (5)$$

Here $\bar{N}_{AM}$ and $\bar{N}_{AATPAC}$ were the average segment length of the monomer AM and AATPAC, respectively. The r_{AM} (CPAA), r_{AATPAC} (CPAA), r_{AM} (TPAA), and r_{AATPAC} (TPAA)

were 2.815, 0.358, 1.586, and 0.751, respectively. $[M_{AM}]$ and $[M_{AATPAC}]$ were the dosages (unit: mol) of AM and AATPAC before the reaction and the mole ratio of $[M_{AM}]$ (60 mmol) to $[M_{AATPAC}]$ (40 mmol) was 1.500.

Table S3. The average segment length of AM and AATPAC in CPAA and TPAA, respectively

Copolymer	$\bar{N}_{AM}$	
CPAT	5.223	1.239
TPAT	3.379	1.501

Table S4

Table S4. The sequence distributions of the monomer segments

X	CPAA		CPAA	
	$(P_1)_x$ (%):AM	$(P_2)_x$ (%):AATPAC	$(P_1)_x$ (%):AM	$(P_2)_x$ (%):AATPAC
1	20.7	80.7	30.1	66.6
2	16.4	15.5	21.0	22.2
3	13.0	3.0	14.7	7.4
4	10.3	0.5	10.3	2.5
5	9.5	0.1	7.1	0.8
6	8.2	···	5.0	0.3
7	6.5	···	3.5	0.1
8	5.1	···	2.4	···
9	4.1	···	1.7	···
10	3.2	···	1.2	···
11	2.6	···	0.8	···
12	2.0	···	0.6	···
13	1.6	···	0.4	···
14	1.3	···	0.3	···
15	1.0	···	0.2	···
16	0.8	···	0.1	···
17	0.5	···	···	···
18	0.4	···	···	···

…	…	…	…	…
sum	100	100	100	100

Table S5

Table S5. The details of the flocculants used in the dewatering test

Abbreviation	Cationic degree	Intrinsic viscosity (dL·g^{-1})	Conversion rate (%)	Synthetic method
TPAA	38.5	4.75	99.3	UTP
CPAA	38.5	4.72	99.5	PT
CPDA	38.5	4.74	/	PT
CPMA	38.5	4.73	/	PT

UTP: ultrasonic-initiated template copolymerization.
PT: polymerization technique without ultrasonic and template.
CPDA and CPMA are commercial flocculants.

The total monomer conversion of polymerization was determined by the gravimetric method [2].

Figure S2

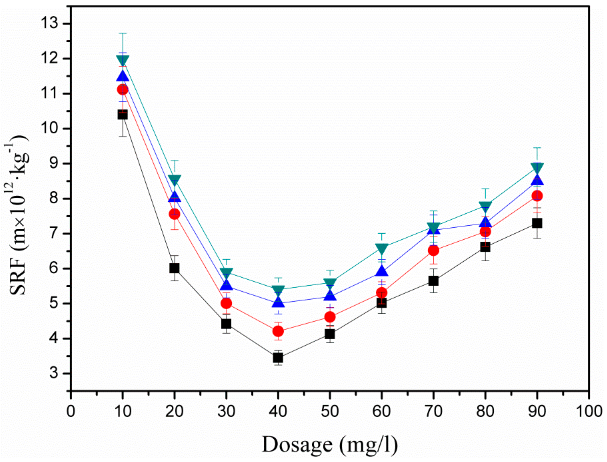

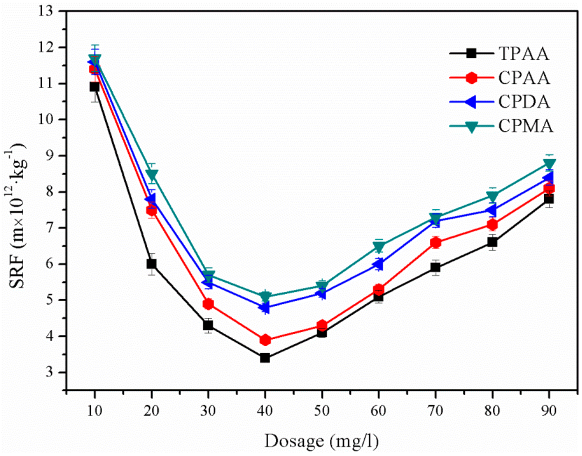

Figure. S2. Effect of the dosage on SRF.

Figure S3

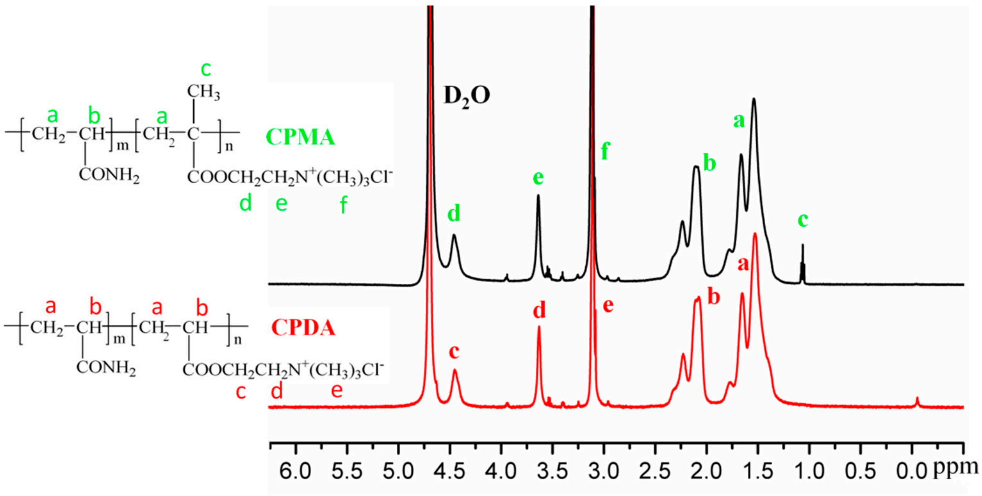

Figure. S3. The ^{1}H NMR spectra of the CPMA and CPDA.

The ^{1}H NMR spectra of CPMA and CPDA are shown in Figure S3 to further analyze their microstructure. The proton adsorption peaks at δ = 1.62 ppm (H_a) and δ = 2.19 ppm (H_b) were derived from the backbone methylene groups $-CH_2-$ and methine groups $-CH-$, respectively, for both CPMA and CPDA. The characteristic proton peaks at δ = 1.16 ppm (H_c), δ = 4.46 ppm (H_d), δ = 3.71 ppm (H_e), δ = 3.19 ppm (H_f) were assigned to the protons of $-CH_3$, $-O-CH_2-$, $-CH_2-N^+$, and $-N^+(CH_3)_3$ in the DMC monomer of CPMA [3]. Meanwhile, the characteristic proton peaks at δ = 4.47 ppm (H_c), δ = 3.72 ppm (H_d), and δ = 3.19 ppm (H_e) were assigned to the protons of $-O-CH_2-$, $-CH_2-N^+$ and $-N^+(CH_3)_3$ in the DAC monomer of CPDA [4]. These results indicated that CPMA and CPDA were successfully prepared.

References

1. Zhang, H.; Yang, J.K.; Yu, W.B.; Luo, S.; Peng, L.; Shen, X.X.; Shi, Y.F.; Zhang, S.N.; et al. Mechanism of red mud combined with Fenton's reagent in sewage sludge conditioning. *WATER Res*, **2014**, 59, 239-247.
2. Niranjan, P. S.; Tiwari, A. K.; Upadhyay, S. K. Polymerization of acrylamide in a micellar medium: Inhibition effect of surfactants. *J.Appl. Polym. Sci*. **2011**, 122, 981-986.
3. Liu, B.Z.; Zheng, H.L.; Deng, X.R.; Xu, B.C.; Sun, Y.J.; Liu, Y.Z.; Liang, J.J. Formation of cationic hydrophobic micro-blocks in P(AM-DMC) by template assembly: characterization and application in sludge dewatering. RSC Adv. **2017**, 7, 6114-6122.
4. Guan, Q.Q.; Zheng, H.L.; Zhai, J.; Zhao, C.; Zheng, X.K.; Tang, X.M.; Chen, W.; Sun, Y.J. Effect of Template on Structure and Properties of Cationic Polyacrylamide: Characterization and Mechanism. *Ind. Eng. Chem. Res*. **2014**, 53, 5624-5635.
